# Creatinine to Cystatin-C Ratio in Renal Cell Carcinoma: A Clinically Pragmatic Prognostic Factor and Sarcopenia Biomarker

**DOI:** 10.1093/oncolo/oyad218

**Published:** 2023-08-04

**Authors:** Benjamin N Schmeusser, Henry Biermann, Edouard H Nicaise, Adil A Ali, Dattatraya H Patil, Eric Midenberg, Talia Helman, Manuel Armas-Phan, Reza Nabavizadeh, Shreyas S Joshi, Vikram M Narayan, Mehmet A Bilen, Sarah P Psutka, Kenneth Ogan, Viraj A Master

**Affiliations:** Department of Urology, Indiana University School of Medicine, Indianapolis, IN, USA; Department of Urology, Emory University School of Medicine, Atlanta, GA, USA; Department of Urology, Emory University School of Medicine, Atlanta, GA, USA; Department of Urology, Emory University School of Medicine, Atlanta, GA, USA; Department of Urology, Emory University School of Medicine, Atlanta, GA, USA; Department of Urology, University of Louisville, Louisville, KY, USA; Department of Urology, Emory University School of Medicine, Atlanta, GA, USA; Department of Urology, Emory University School of Medicine, Atlanta, GA, USA; Department of Urology, Mayo Clinic, Rochester, MN, USA; Department of Urology, Mayo Clinic, Rochester, MN, USA; Department of Urology, Mayo Clinic, Rochester, MN, USA; Department of Hematology and Medical Oncology, Emory University School of Medicine, Atlanta, GA, USA; Department of Urology, University of Washington, Seattle, WA, USA; Department of Urology, Fred Hutchinson Cancer Center, Seattle, WA, USA; Department of Urology, Emory University School of Medicine, Atlanta, GA, USA; Department of Urology, Emory University School of Medicine, Atlanta, GA, USA

**Keywords:** renal cell carcinoma (RCC), nephrectomy, survival, sarcopenia, body composition, biomarker

## Abstract

**Introduction:**

Low creatinine to cystatin-C ratio (Cr/Cys-C) may be a biomarker for low-muscle mass. Furthermore, low Cr/Cys-C is associated with decreased overall survival (OS), but to date, has not been examined in patients with renal cell carcinoma (RCC). Our objective is to evaluate associations between low Cr/Cys-C ratio and OS and recurrence-free survival (RFS) in patients with RCC treated with nephrectomy.

**Methods:**

We performed a retrospective review of patients with RCC treated with nephrectomy. Patients with end-stage renal disease and less than 1-year follow up were excluded. Cr/Cys-C was dichotomized at the median for the cohort (low vs. high). OS and RFS for patients with high versus low Cr/Cys-C were estimated with the Kaplan-Meier method, and associations with the outcomes of interest were modeled using Cox proportional Hazards models. Associations between Cr/Cys-C and skeletal muscle mass were assessed with correlations and logistic regression.

**Results:**

A total of 255 patients were analyzed, with a median age of 64. Median (IQR) Cr/Cys-C was 1 (0.8-1.2). Low Cr/Cys-C was associated with age, female sex, Eastern Cooperative Oncology Group Performance Status ≥1, TNM stage, and tumor size. Kaplan-Meier and Cox regression analysis demonstrated an association between low Cr/Cys-C and decreased OS (HR = 2.97, 95%CI, 1.12-7.90, *P* =0.029) and RFS (HR = 3.31, 95%CI, 1.26-8.66, *P* = .015). Furthermore, a low Cr/Cys-C indicated a 2-3 increase in risk of radiographic sarcopenia.

**Conclusions:**

Lower Cr/Cys-C is associated with inferior oncologic outcomes in RCC and, pending validation, may have utility as a serum biomarker for the presence of sarcopenia in patients with RCC treated with nephrectomy.

## Teaser

Low creatinine to cystatin-C ratio (Cr/Cys-C) is associated with decreased overall survival (OS) but, to date, has not been examined in patients with renal cell carcinoma (RCC). This article evaluates associations between low Cr/Cys-C ratio and OS and recurrence-free survival in patients with RCC treated with nephrectomy.

**Implications for Practice**

Creatinine and cystatin-C are 2 routine markers of kidney function obtained from blood samples. A ratio of creatinine to cystatin-C may offer a clinically practical method to identify patients with low muscle and at risk for worse survival in kidney cancer.

## Introduction

Despite improvements in detection and advancements in therapeutics for renal cell carcinoma (RCC), 5-year survival rates remain around 75%.^1^ Currently, prognostication of RCC relies heavily on a combination of anatomical and pathological features.^2^ While informative, predictive models that combine commonly used risk factors with readily available and patient-specific pretreatment data to further direct clinical management are needed.^2,3^

Sarcopenia, or decreased muscle mass, has emerged as a significant prognostic factor for worse survival across solid-organ malignancies,^4-6^ including RCC.^7-12^ However, widespread clinical adoption of body composition analysis is limited by constraints, such as money, time, training, and the availability of specialized software to obtain detailed measurements of muscle and fat mass.^13,14^ As such, readily available and low-cost biomarkers for low muscle mass to identify patients with sarcopenia are needed.

One candidate that may assist in the detection of sarcopenia is the creatinine (Cr) to cystatin-C (Cys-C) ratio (Cr/Cys-C). Because Cr is produced by muscle cells and Cys-C is produced by all nucleated cells, a lower Cr/Cys-C is considered a surrogate of low muscularity. Cr/Cys-C is especially attractive given that both laboratory values are widely available serum tests that could be efficiently scaled to increase the identification of low muscularity and high-risk patients more effectively.

The association and correlation of Cr/Cys-C with muscle composition have been demonstrated in various populations, including patients with malignancy.^15-23^ Furthermore, Cr/Cys-C is associated with overall survival in malignancies, such as lung and esophageal cancer.^19-21,24^ While literature demonstrates that Cr/Cys-C is prognostic in various patient populations, its utility has not been examined in patients with RCC exclusively. Examination of the Cr/Cys-C in the RCC population is particularly intriguing given the optimal performance of combined Cr- and Cys-C-based renal function equations^25^ and the importance of accurate renal function assessment for patients undergoing nephrectomy and other treatments.

Here, we first assessed whether Cr/Cys-C was prognostic of overall survival (OS) and recurrence-free survival (RFS) after partial or radical nephrectomy in patients with RCC. We also evaluated the association between Cr/Cys-C and muscle mass. We hypothesized that lower Cr/Cys-C would be prognostic of worse RFS and OS and that it would also be significantly associated with lower muscle mass.

## Methods

### Patient Selection

**Figure 1.**
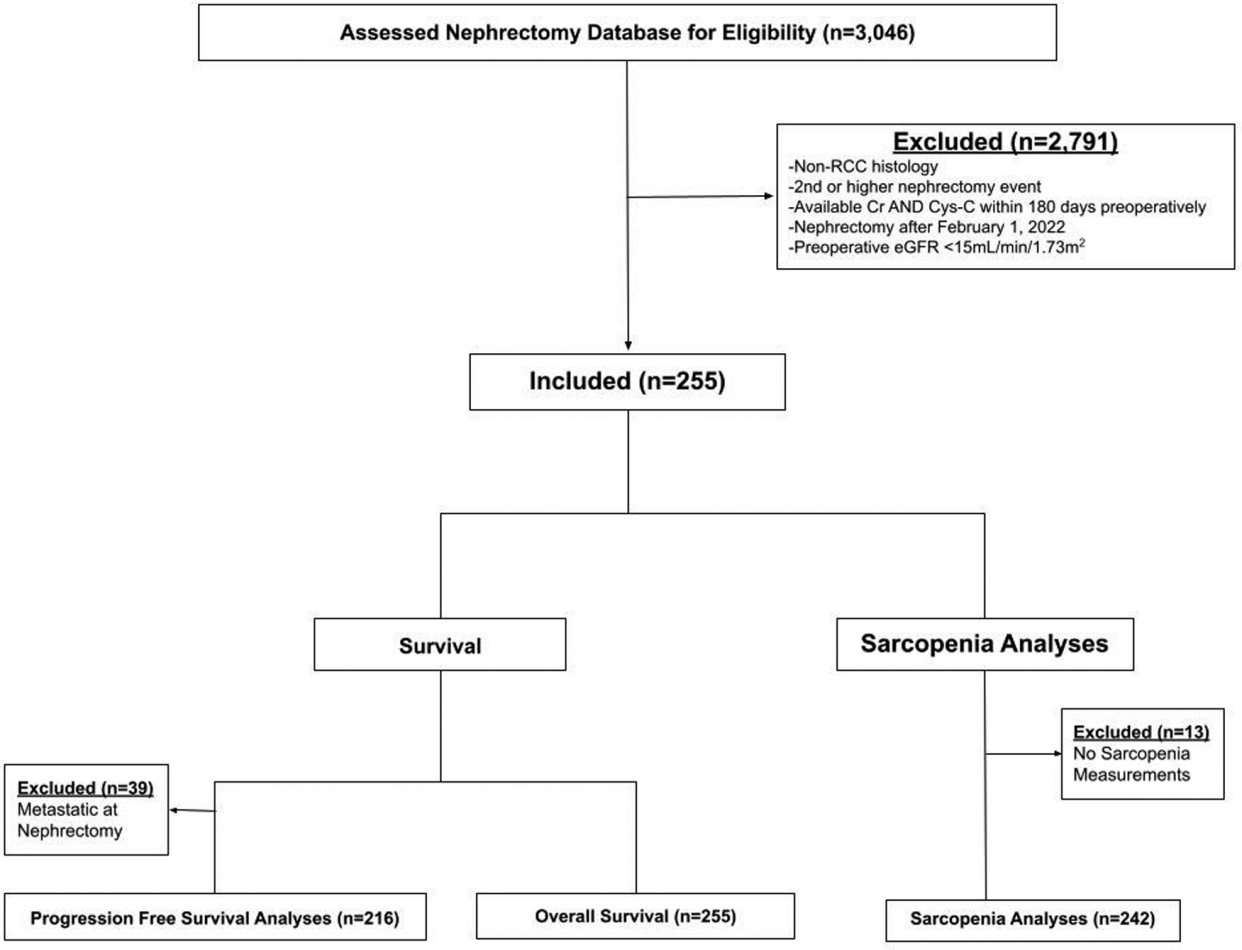
Study flow chart for inclusion/exclusion.

Figure 1 presents the flow chart for study inclusion and exclusion. This is a retrospective analysis of a prospectively maintained database examining patients who underwent partial or radical nephrectomy for RCC at a single tertiary referral center from 2018 to 2022. Included were all patients with RCC who underwent a definitive surgical intervention, had a serum Cr and Cys-C completed at most 6 months prior to surgery, and had nephrectomy occurring at least 1 year prior to analysis. Patients with eGFR <15 mL/minute/1.73 m^2^ were excluded, as determined by the Chronic Kidney Disease Epidemiology Collaboration (CKD-EPI) 2021 Cr equation (CKD-EPI2021). Preoperative patient-specific variables included age, gender, race, body mass index (BMI; kg/m^2^), Eastern Cooperative Oncology Group (ECOG) score, and history of diabetes, hypertension, and cardiovascular disease were included as covariates. Renal function was determined by both the CKD-EPI2021 equation and the CKD-EPI Cys-C equation (CKD-EPI-CysC). Postoperative tumor-specific variables included tumor, node, metastasis (TNM) staging; Fuhrman grade; histology; necrosis; and tumor size, as determined by the largest tumor diameter recorded in the pathology report. The study received approval from the Institutional Review Board.

### Calculating Skeletal Muscle Index and Defining Sarcopenia

In this study, sarcopenia refers to radiographic/anatomic sarcopenia. Preoperative axial CT or MRI images were segmented at the mid-level of the L3 vertebral body, per validated methodology.^10,26^ Images obtained from both imaging modalities were used for the analysis, as previous studies have shown a strong correlation between CT and MRI modalities for accurately measuring body composition parameters.^13^ Images were analyzed by observers trained in segmentation with <1% interindividual variability. Observers were blinded to clinical outcomes. Slice-O-Matic software (version 5.0; Tomovision) was used to quantify the L3 skeletal muscle area (SMA) in cm² using a predesignated threshold of −29 to +150 Hounsfield units on CT imaging; for MRI, the region growing tool was used.^13,27^ Determination of the total L3 cross-sectional muscle area involved the summation of SMA measurements from the psoas major, quadratus lumborum, erector spinae, and abdominal wall musculature (rectus abdominus, tranversus abdominus, external oblique, and internal oblique).^7,9,28,29^ The skeletal muscle index (SMI) was calculated by normalizing the SMA by the square of the height in square meter.

In this study, sarcopenia was categorized using 2 sex-specific definitions (Supplementary Table S1). First, sarcopenia was defined by the sex- and BMI-based Martin et al criteria,^26^ which has been widely validated in patients with RCC.^12,30,31^ Under these criteria, sarcopenia in males is defined as SMI <53 cm^2^/m^2^ if BMI >25 kg/m^2^ or SMI <43 cm^2^/m^2^ if BMI <25 kg/m^2^. For women, sarcopenia was defined as <41 cm^2^/m^2^. Furthermore, given that sarcopenia thresholds may vary in more heterogeneous populations with different races and ethnicities, thresholds fit to our patient population (Emory criteria) were used, as previously published.^9,10,32^ These optimally fit sex- and BMI-stratified SMI thresholds were determined by receiver operating characteristic (ROC) analysis and grid search best-fit method, similar to other studies.^9,26,28,33^ The Emory sarcopenia criteria were defined, as an SMI <47 cm^2^/m^2^ for males and <38 cm^2^/m^2^ for females in patients with a BMI <30 kg/m^2^. For patients with a BMI ≥30 kg/m^2^, male and female patients were classified as sarcopenic, if SMI <54 cm^2^/m^2^ or <47 cm^2^/m^2^, respectively.

### Statistical Analysis

The primary outcomes of interest were OS and RFS, and the secondary outcome was the presence of sarcopenia at the time of surgery, while primary exposure was Cr/Cys-C. Sarcopenia was defined by Martin and Emory criteria, as detailed earlier. OS was defined as the time from surgery to death from any cause or day of last follow up recorded in patient charts. RFS was defined as time from surgery to recurrence of RCC in patients nonmetastatic at the time of surgery, confirmed by either radiological or histological evidence.

The cohort was dichotomized at median Cr/Cys-C to categorize patients as having high versus low Cr/Cys-C. Patient and clinical characteristics were compared between both groups using a generalized chi-square test or Fisher’s exact test for categorical variables and a Wilcoxon rank-sum test for continuous variables.

Survival analysis was first conducted comparing both groups in terms of the primary outcomes using the Kaplan-Meier method. In addition, multivariable Cox proportional hazards regression models with stepwise selection for OS and RFS were fit using Cr/Cys-C ratio (binary and continuous), age at surgery, sex, race, ECOG status, obesity (BMI >30 kg/m^2^), and diabetes as a priori selected variables. After collinearity assessments, M-stage, systemic treatment, and RCC histology were additionally included in the OS model, and type of nephrectomy, T-stage, N-stage, RCC histology, Fuhrman grade, necrosis, and maximum tumor width were included in the RFS model. Due to evidence that elevated Cys-C alone may have some prognostic value in patients with RCC, a separate analysis of Cys-C only as a predictor was independently conducted.^34,35^

To provide further context for the prognostic ability of Cr/Cys-C ratio in this cohort, the UCLA Integrated Staging System (UISS)^36^ and an updated RCC prognostic scoring system reported in 2018 by Leibovich et al,^37^ referred to as Mayo Prognostic Scoring System (MPS) in this article, were additionally examined in this cohort. AUC analysis for RFS at 12 months postoperatively was conducted for Cr/Cys-C, MPS, and MPS + Cr/Cys-C. For the UISS, trends in Cr/Cys-C based on low-intermediate-high risk grouping for nonmetastatic or metastatic disease were analyzed. Finally, we identified patients with metastatic disease and, in patients with available data, calculated the international metastatic RCC database consortium (IMDC) and Memorial Sloan Kettering Cancer Center (MSKCC) risk scores to examine associations of Cr/Cys-C with favorable-to-intermediate risk and poor risk disease.

Next, Spearman’s Rank-Order correlation was first used to determine the relationship between Cr/Cys-C and SMI, with BMI stratified subanalysis (20-25 kg/m^2^ and >25 kg/m^2^ given the difference in our cohort BMI (~29 kg/m^2^) and the BMIs reported in most of the Cr/Cys-C literature (22-27 kg/m^2^).^15-21,23,38^ Then, ROC and multivariable logistic regression models were constructed to evaluate association between Cr/Cys-C and sarcopenia. Using low Cr/Cys-C, age >65, gender, ECOG PS, histologic type, BMI, and TNM stage, multivariable models were then fit with stepwise selection for sarcopenia as an outcome (defined by both Emory and Martin criteria).

All statistical tests were 2-sided with type I error set at 0.05. All analyses were performed using SAS version 9.4 (Cary, NC, USA).

## Results

### Cohort Summary

Study cohort demographics and tumor characteristics are displayed in Table 1. The cohort consisted of 255 patients with RCC who underwent surgery and met inclusion criteria between 2018 and 2022. With the CKD-EPI2021 Cr-based equation, 75.7%, 22.4%, and 2.0% of patients had CKD stages I-II, III, and IV, respectively. When using the CKD-EPI-CysC equation, 63.5%, 31.4%, and 5.1% of patients had CKD stages I-II, III, and IV, respectively. Median (IQR) Cr was 1.1 mg/dL (0.9-1.3 mg/dL) and Cys-C level was 1.0 mg/L (0.8-1.3). The median (IQR) Cr/Cys-C was 1.0 (0.8-1.2). Most patients underwent radical nephrectomy (61.6%) for clear cell RCC (71.4%). Patients (44.5%) had T1-T2 and 54.5% had T3-T4 staged disease. Patients (11.8%) had nodal involvement, and 15.3% had evidence of metastatic disease.

**Table 1.** Descriptive cohort (*n* = 255) by above or below preoperative creatinine/cystatin-c ratio median of 1.0 (0.8-1.2).

	*n* (%)	Median (IQR) Cr/Cys-C	Cr/Cys-C < 1 (*n*%)	Cr/Cys-C > 1 (*n*%)	*P*-value
Age, years^*^	64 (54-72)	–0.192^**^	65.3 (56.4-73)	62.8 (53.7-70)	.025
Gender					
Male	171 (67.1)	1.1 (0.9-1.2)	52 (47.7)	119 (81.5)	<.001
Female	84 (32.9)	0.8 (0.7-1.0)	57 (52.3)	27 (18.5)	
Race					
White	161 (63.1)	1.0 (0.8-1.1)	78 (71.6)	83 (56.8)	.024
Black	69 (27.1)	1.1 (0.9-1.3)	20 (18.3)	49 (33.6)	
Other	25 (9.8)	1.0 (0.9-1.1)	11 (10.1)	14 (9.6)	
ECOG					
0	230 (90.2)	1.0 (0.8-1.2)	92 (84.4)	138 (94.5)	.007
≥ 1	25 (9.8)	0.9 (0.7-1.0)	17 (15.6)	8 (5.5)	
BMI (kg/m^2^)^*^	28.9 (25.8-34)	-0.1^**^	29.3 (25.8-35.9)	28.7 (25.8-33.4)	.305
Diabetes					
Yes	88 (34.5)	1.0 (0.8-1.2)	42 (38.5)	46 (31.5)	.243
No	167 (65.5)	1.0 (0.8-1.2)	67 (61.5)	100 (68.5)	
Hypertension					
Yes	207 (81.2)	1.0 (0.8-1.2)	91 (83.5)	116 (79.5)	.415
No	48 (18.8)	1.0 (0.8-1.3)	18 (16.5)	30 (20.5)	
Cardiovascular disease					
Yes	67 (26.3)	1.0 (0.8-1.2)	31 (28.4)	36 (24.7)	.497
No	188 (73.7)	1.0 (0.8-1.2)	78 (71.6)	110 (75.3)	
Preoperative renal function (CKD-EPI-Cr-2021)					
eGFR^*^	72 (60-90)	−0.111^**^	74 (60-92)	71 (59-87)	.322
CKD Stages I-II	193 (75.7)	1.0 (0.8-1.2)	84 (77.1)	109 (74.7)	.573
CKD Stage III	57 (22.4)	1.0 (0.8-1.2)	24 (22)	33 (22.6)	
CKD Stage IV	5 (2)	1.1 (1.0-1.1)	1 (0.9)	4 (2.7)	
Preoperative renal function (CKD-EPI-Cys-C))					
eGFR^*^	71 (49-94)	0.543^**^	53 (40-74)	87 (65-104)	<.001
CKD Stages I-II	162 (63.5)	1.1 (0.9-1.3)	46 (42.2)	116 (79.5)	<.001
CKD Stage III	80 (31.4)	0.9 (0.7-1.0)	54 (49.5)	26 (17.8)	
CKD Stage IV	13 (5.1)	0.8 (0.7-1.0)	9 (8.3)	4 (2.7)	
Type of nephrectomy					
Radical	157 (61.6)	0.9 (0.8-1.2)	79 (72.5)	78 (53.4)	.002
Partial	98 (38.4)	1.1 (0.9-1.3)	30 (27.5)	68 (46.6)	
Pathologic T-stage					
T1-T2	116 (45.5)	1.1 (0.9-1.3)	36 (33)	80 (54.8)	<.001
T3-T4	139 (54.5)	0.9 (0.7-1.1)	73 (67)	66 (45.2)	
Pathologic N-stage					
N0	161 (63.1)	1.0 (0.8-1.2)	69 (63.3)	92 (63)	.993
N1	30 (11.8)	1.0 (0.7-1.1)	13 (11.9)	17 (11.6)	
NX	64 (25.1)	1.0 (0.8-1.2)	27 (24.8)	37 (25.3)	
Pathologic M-stage					
M0	216 (84.7)	1.0 (0.9-1.2)	83 (76.1)	133 (91.1)	.001
M1	39 (15.3)	0.8 (0.7-1.0)	26 (23.9)	13 (8.9)	
Histology					
Clear Cell	182 (71.4)	1.0 (0.8-1.1)	84 (77.1)	98 (67.1)	.136
Papillary	27 (10.6)	1.2 (1.0-1.3)	6 (5.5)	21 (14.4)	
Chromophobe	24 (9.4)	1.1 (0.8-1.3)	10 (9.2)	14 (9.6)	
Other	22 (8.6)	1.1 (0.8-1.2)	9 (8.3)	13 (8.9)	
Necrosis					
No	142 (55.7)	1.0 (0.9-1.2)	53 (48.6)	89 (61)	.05
Yes	113 (44.3)	1.0 (0.8-1.2)	56 (51.4)	57 (39)	
Furhman grade (missing = 29)					
G1-G2	39 (17.3)	1.1 (0.9-1.3)	12 (12.4)	27 (20.9)	.092
G3-G4	187 (82.7)	1.0 (0.8-1.2)	85 (87.6)	102 (79.1)	
Tumor > 7cm					
Yes	112 (43.9)	0.9 (0.7-1.1)	50 (45.9)	93 (63.7)	.005
No	143 (56.1)	1.1 (0.9-1.2)	40 (43.5)	78 (61.4)	

^*^Median (IQR).

^**^Pearson correlation coefficient for continuous variables.

Abbreviations: ECOG: Eastern Cooperative Oncology Group; BMI: body mass index; CKD-EPI-Cr-2021: Chronic Kidney Disease Epidemiology Collaboration 2021 Creatinine Equation without Race; CKD-EPI-Cys-C: Chronic Kidney Disease Epidemiology Collaboration Cystatin C Equation; eGFR: estimated glomerular filtration rate; SMI: skeletal muscle index.

Patients with low versus high Cr/Cys-C ratio differed such that patients with a low Cr/Cys-C ratio were more likely to be older, female, with ECOG PS ≥1, and higher CKD stage as determined by CKD-EPI-CysC eGFR equation. With respect to pathologic characteristics, low Cr/Cys-C was associated with increased prevalence of pT3/4 disease, M1 disease, Tumor size > 7cm and risk of recurrence (Table 1).

### Cr/Cys-C Ratio and Survival Outcomes

**Figure 2.**
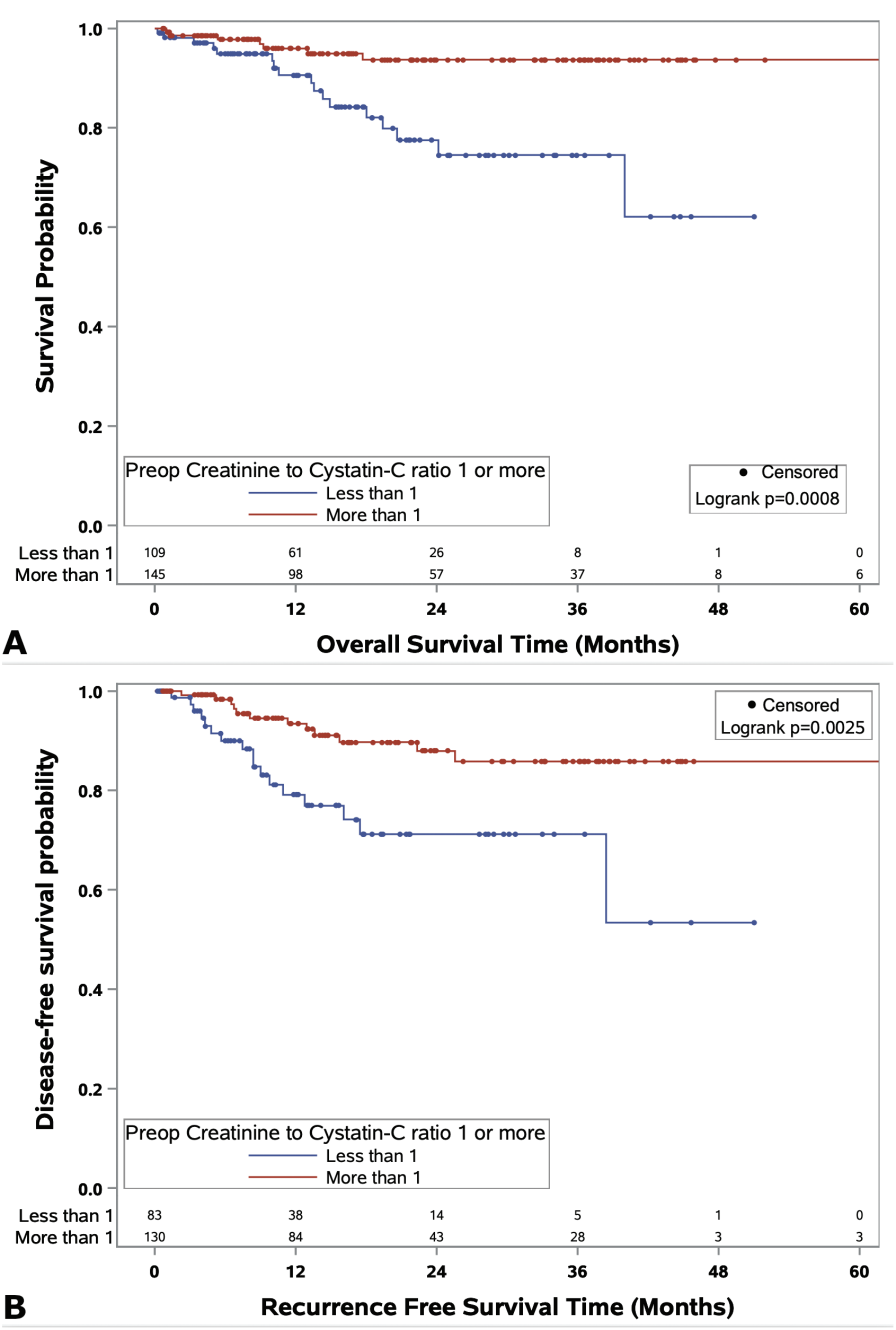
(**A**) Kaplan-Meier curve displaying overall survival for patients with above or below median (1.0) creatinine to cystatin-c ratio (*n* = 255). (**B**) Kaplan-Meier curve displaying recurrence-free survival for patients with above or below median (1.0) creatinine to cystatin-c ratio (*n* = 216, nonmetastatic).

Median follow-up for survivors was 16.6 months, during which 48 patients developed recurrence and 25 patients died of any cause. Fig. 2 displays the results from Kaplan-Meier analyses for low versus high Cr/Cys-C for the outcomes of OS and RFS. At 24 months, the median OS was 77.5% for low Cr/Cys-C and 93.5% for high Cr/Cys-C (Fig. 2A, *P* = .0008). Similarly, 71.8% versus 88.0% of patients with a low versus a high Cr/Cys-C, respectively, were recurrence-free at 24 months (Fig. 2B, *P* = .0025).

Table 2 summarizes the results from multivariable Cox proportional hazards regression analysis for OS and RFS. After adjusting for gender, obesity, diabetes, grade, T-stage, and N-stage, low Cr/Cys-C was independently predictive of worse overall survival (HR = 2.97, 95%CI, 1.12-7.90, *P* = .029). Furthermore, when analyzing Cr/Cys-C as a continuous variable and adjusting for age, gender, ECOG, obesity, diabetes, histology, grade, N-stage, and tumor size, a 0.1 increase in the Cr/Cys-C ratio was associated with an approximately 23% decrease in the risk of death (HR = 0.77, 95%CI, 0.61-0.97). Full results from OS multivariable analysis are displayed in Supplementary Table S2. Regarding RFS, after adjustment for age, gender, race, histology, ECOG, N-stage, necrosis, nephrectomy type, obesity, T-stage, necrosis, and tumor width, low Cr/Cys-C ratio was independently associated with risk of recurrence (HR = 3.31, 95%CI, 1.26-8.66), along with the presence of necrosis (HR = 3.71, 95%CI, 1.24-11.11). Similarly, in the continuous model, a 0.1 increase in the Cr/Cys-C ratio was associated with an approximately 23% decrease in risk of recurrence (HR = 0.77, 95%CI, 0.63-0.96). Full results from RFS multivariable analysis are presented in Supplementary Table S3.

**Table 2.** Multivariable Cox proportional hazards regression analyses for predictive ability of creatinine/cystatin-c ratio on overall survival (*n* = 255) and recurrence-free survival (*n* = 216, nonmetastatic only).

	Overall survival		Recurrence-free survival	
	Hazard ratio (95% CI)	*P*-value	Hazard ratio (95% CI)	*P*-value
Low creatinine to cystatin-C ratio	2.97 (1.12-7.90)	.029	3.31 (1.26-8.66)	.015
Continuous creatinine to cystatin-C ratio	0.77 (0.61-0.97)	.024	0.77 (0.63-0.96)	.018

Overall survival: Multivariable COX proportional stepwise final model evaluating creatinine to cystatin-C ratio as a binary variable (above or below median) was simultaneously adjusted for gender, obesity, Fuhrman grade, T-stage, and N-stage. Multivariable COX proportional stepwise final model evaluating creatinine to cystatin-C ratio as a continuous variable was simultaneously adjusted for age, gender, ECOG status, obesity, diabetes, histology, Fuhrman grade, N-stage, and maximum tumor width (Supplementary Table S2).

Recurrence-free survival: Multivariable COX proportional stepwise model evaluating creatinine to cystatin-C ratio as a binary variable (above or below median) was simultaneously adjusted for age >65, Black race, gender, clear cell histology, ECOG status, N-stage, necrosis, nephrectomy type, obesity, T-stage, necrosis, and tumor width. As a continuous variable, the model was adjusted for the same factors as binary variable with additional inclusion of diabetes (Supplementary Table S3).

A separate analysis was conducted to determine the effects of Cys-C alone. Supplementary Table S4 displays Cys-C distribution by patient characteristics, with multivariable analysis (Supplementary Table S5) finding continuous Cys-C (unit = 0.1 mg/L) having some prognostic ability for OS (HR = 1.12, 95%CI, 1.01-1.24, *P* = .032) and RFS (HR = 1.09, 95%CI, 1.09-1.19, *P* = .03).

### Performance in Relation to Established RCC Prognostic Models

**Figure 3.**
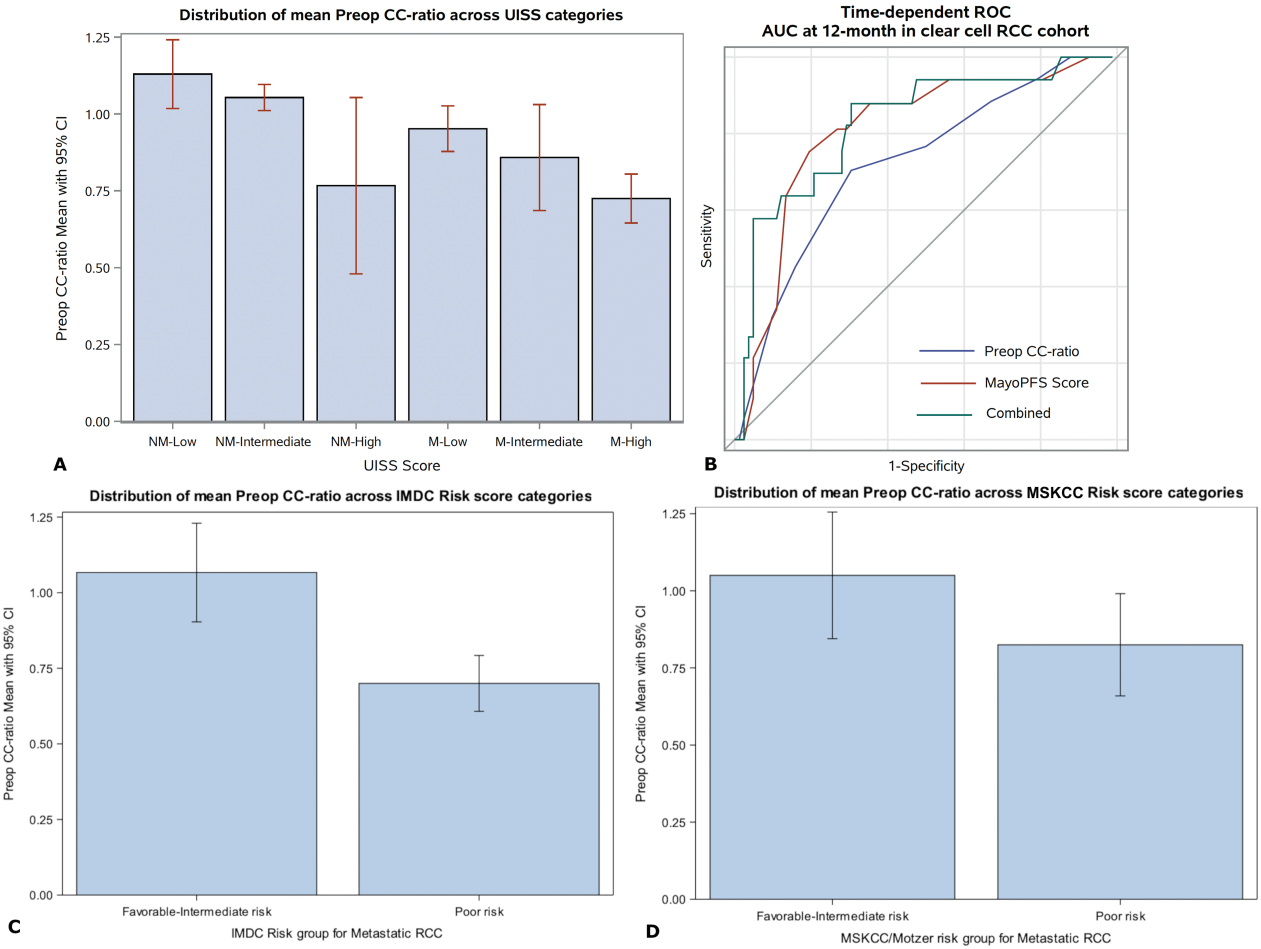
Cr/Cys-C ratio in relation to UCLA Integrated Staging System (UISS) and Mayo Prognostic Scoring System (MPS). (**A**) Trends in Cr/Cys-C ratio based on UISS risk scores, revealing a decrease in mean Cr/Cys-C ratio as risk grouping increases for both nonmetastatic (NM) and metastatic (M) disease. (**B**) Area under the curve analysis at 12 months for Cr/Cys-C ratio, MPS, and MPS + Cr/Cys-C ratio. (**C**) and (**D**) Distribution of mean preoperative Cr/Cys-C ratios across favorable-intermediate versus poor IMDC and MSKCC risk score categories.

Cr/Cys-C ratio in relation to the UISS, MPS, IMDC, and MSKCC risk scores is presented in Fig. 3. In relation to UISS (Fig. 3A), statistically significant decreases (*P* < .001) in Cr/Cys-C was observed as UISS risk category increased. For example, median (IQR) for nonmetastatic low, intermediate, and high risk was 1.1 (0.7-2.1), 1.0 (0.5-1.6), and 0.7 (0.7-0.9), respectively. On ROC analysis for MPS (Fig. 3B), the AUC 0.723 for Cr/Cys-C ratio, 0.807 for MPS, and 0.827 for MPS with Cr/Cys-C ratio. Of the 39 patients with metastatic disease, only 16 and 12 patients had sufficient data for IMDC and MSKCC risk score calculations, respectively. One hundred percent (*n* = 7) of patients with poor risk IMDC scores and 88% (*n* = 7) of the patients with poor risk MSKCC scores had a Cr/Cys-C ratio less than 1. Furthermore, comparing poor risk versus favorable-to-intermediate risk scores, median (IQR) Cr/Cys-C ratio was significantly lower using IMDC (0.7 [0.7-0.8] vs. 1.1 [0.9-1.2], *P* < .001, Fig. 3C) and lower, though nonsignificant, using MSKCC (0.8 [0.7-0.9] vs. 1.1 [1.0-1.2], *P* = .07, Fig. 3D). With recognition of the significantly limited sample size, results trend toward lower Cr/Cys-C ratios in patients with poor risk metastatic scores.

### Relationship Between Cr/Cys-C Ratio and Sarcopenia

Two hundred and forty two out of the 255 patients had sufficient imaging for SMI assessment, with a median of 36 (Q1-Q3: 16-65) days between imaging and nephrectomy. As displayed in Table 3, the median SMI was 49.9 cm^2^/m^2^ for males and 39.9 cm^2^/m^2^ for females. Based on the Emory and Martin sarcopenia thresholds, 129 (53.3%) and 140 (46.7%) of the patients, respectively, were classified as sarcopenic. Patients with low Cr/Cys-C had significantly lower SMI (39.0 cm^2^/m^2^ vs. 43.0 cm^2^/m^2^ for females and 43.6 cm^2^/m^2^ vs. 51.3 cm^2^/m^2^ for males; Supplementary Fig. S1). Furthermore, rates of sarcopenia for patients were significantly higher for patients with low Cr/Cys-C compared to high Cr/Cys-C for both Martin (48.6% vs. 35.3%, *P* = .039) and Emory thresholds (54.3% vs. 30.1%, *P* < .001). An opposite trend was observed for patients with high Cr/Cys-C, finding a significantly lower proportion of patients being considered sarcopenic by both Martin (51.4% vs. 64.7%, *P* = .39) and Emory thresholds (45.7% vs. 69.9%, *P* < .001).

**Table 3.** Descriptive summary of SMI and sarcopenia (*n* = 242) in relationship to creatinine to cystatin-C ratio.

		Cr/Cys-C < 1	Cr/Cys-C > 1	*P*-value
SMI (cm^2^/m^2^])^*^				
Males	49.9 (41.9-56.2)	43.6 (37.0-53.9)	51.3 (44.8-57.3)	<.001
Females	39.9 (34.8-49.1)	39.0 (32.3-48.5)	43 (37.3-49.1)	<.001
Sarcopenic (*n*%)				
Yes (Emory)	129 (53.3)	70 (54.3)	59 (45.7)	<.001
No (Emory)	113 (46.7)	34 (30.1)	79 (69.9)	
Yes (Martin)	140 (57.9)	68 (48.6)	72 (51.4)	.039
No (Martin)	102 (42.1)	36 (35.3)	66 (64.7)	

^*^Median (IQR).

Abbreviation: SMI: skeletal muscle index.

Correlations between Cr/Cys-C and SMI are displayed in Supplementary Table 6. For men, Cr/Cys-C was positively and significantly correlated with SMI (0.229, *P* < .001); however, for women, this relationship trended toward but was not significant (0.203, *P* = .075). Notably, a correlation between SMI and Cr/Cys-C ratio was observed when stratifying by normal BMI versus overweight BMI; however, the relationship was stronger for individuals with normal body weight (ie, 0.506 vs. 0.283 SMI correlation for normal weight vs. overweight, respectfully).

To determine the accuracy of low vs. high Cr/Cys-C to reflect sarcopenia, ROC analysis and multivariable logistic regression were conducted. The area under the curve (AUC, 95% CI) for low versus high Cr/Cys-C ratio identification of sarcopenia was 0.634 (0.564-0.704) and 0.605 (0.533-0.677) for the Emory and Martin thresholds, respectively. As shown in Table 4, patients with low Cr/Cys-C were more likely to be sarcopenic with both the Emory (OR 3.1, 95%CI, 1.6-6.1; *P ≤* .001) and Martin (OR 2.1, 95%CI, 1.0-4.3; *P*-value = .031), respectively, after adjusting for age, gender, ECOG PS, BMI, histologic type, T-stage, N-stage, and metastasis.

**Table 4.** Multivariable logistic regression analysis for ability of creatinine to cystatin-C ratio (below median of 1.0) to predict sarcopenia utilizing Emory and Martin sarcopenia cutoffs.

	Emory		Martin	
	OR (95%CI)	*P*-value	OR (95% CI)	*P*-value
Cr/Cys-C < 1.0	3.14 (1.62-6.08)	<.001	2.11 (1.07-4.17)	.031
Age > 65	3.03 (1.66-5.52)	<.001	5.28 (2.81-9.90)	<.001
Female	1.81 (0.89-3.68)	.099	1.11 (0.55-2.24)	.778
ECOG > 1	1.23 (0.44-3.44)	.688	1.34 (0.47-3.83)	.590
BMI (kg/m^2^)				
>35	Ref	<.001	Ref	<.001
25-35	3.43 (1.56-7.54)		6.43 (2.83-14.62)	
<25	7.00 (2.49-19.67)		4.95 (1.82-13.51)	
Clear cell histology	0.77 (0.39-1.51)	.445	0.98 (0.49-1.93)	.944
T-stage				
T1-T2	Ref	Ref	Ref	Ref
T3-T4	0.84 (0.42-1.68)	.622	0.72 (0.35-1.46)	.361
N-stage				
N0	Ref	.01	Ref	.129
N1	1.89 (0.68-5.29)		1.98 (0.70-5.57)	
Nx	0.39 (0.20-0.79)		0.61 (0.30-1.22)	
M-stage	0.71 (0.29-1.76)	.462	1.02 (0.41-2.55)	.970

Abbreviations: ECOG: Eastern Cooperative Oncology Group; BMI: body mass index.

## Discussion

In this study, we assessed the clinically practical Cr/Cys-C ratio in patients with RCC and its association with survival outcomes and sarcopenia. As a continuous measure and as dichotomized into low versus high at the median for this cohort, low Cr/Cys-C for this retrospective cohort was independently associated with worse RFS and OS after adjusting for relevant demographic and tumor-related confounding factors. The prognostic ability of Cr/Cys-C was maintained when examined as a continuous variable for OS (HR = 0.77) and RFS (HR = 0.77). Finally, Cr/Cys-C was positively correlated with SMI, and low Cr/Cys-C was highly correlated with the presence of sarcopenia as categorized by both sex-specific consensus and institution-specific classification systems.

The ability of Cr/Cys-C to prognosticate overall survival has been demonstrated in various oncologic populations. In over 500 patients with advanced non-small cell lung cancer, low Cr/Cys-C was independently prognostic of inferior overall survival.^20^ Similarly, in patients with esophageal cancer, lower Cr/Cys-C was predictive of worse overall survival as well as overall higher surgical complication rates.^19^ Finally, in an analysis of greater than 3000 patients with various malignancies (including 500+ unspecified genitourinary malignancies), a lower Cr/Cys-C was associated with significantly worse 6-month and 1-year overall survival, as well as with longer hospital stays.^21^ Although oncological considerations are emphasized here, the prognostic utility of Cr/Cys-C extends beyond malignancy and has been demonstrated in a variety of acute and chronic non-cancerous conditions, such as in patients with non-dialysis CKD, coronary artery disease, and chronic obstructive pulmonary disease.^39-41^

A primary hypothesis for the Cr/Cys-C ratio’s prognostic ability is that it may serve as a surrogate for sarcopenia or severe muscle mass deficiency due to Cr being produced by skeletal muscle and Cys-C being produced by all nucleated cells, independent of muscle mass.^42,43^ The association of low Cr/Cys-C with sarcopenia has been well demonstrated. For example, Shi et al^38^ demonstrated in over ,500 adults from the NHANES database that low Cr/Cys-C was significantly associated with various sarcopenia definitions and muscle mass as measured by dual-energy X-ray absorptiometry. Similarly, in cohorts of patients with gastric and esophageal cancer, Cr/Cys-C is correlated with SMI (linear correlations of 0.221-0.495) and lower Cr/Cys-C accurately identified patients with sarcopenia.^18,19^ These findings have been replicated in several other studies across malignant and nonmalignant cohorts.^15-21,23,38^ While our results agree with these previous studies, our cohort is unique in that it consists of patients with RCC exclusively, is a diverse population with 30% identifying as Black, and has a higher proportion of overweight and obesity compared to prior studies (median: 29 kg/m^2^ vs. 22-27 kg/m^2^). Interestingly, correlation between Cr/Cys-C and SMI was not statistically significant in our female patients, although the association has previously been established.^44^ The lack of significant correlation with SMI in our cohort could be a result of sample size given, and there were only 84 (32.9%) total females. In addition to, the relatively low, homogeneous distribution of Cr/Cys-C for females as compared to males (females: 0.8, IQR 0.7-1.1; males: 1.1, IQR 0.9-1.2; *P* < .001) likely highlights sex-specific differences in muscle mass. Future large studies determining optimal Cr/Cys-C cutoffs using sex and population-specific consensus definitions with consistent imaging methods in large, diverse populations are warranted.

While this is the first assessment of Cr/Cys-C in an RCC-specific cohort, sarcopenia itself in RCC has been established as an independent predictor of worse OS^7,11,45-50^ and worse CSS.^7,30,45,46^ For example, Psutka et al^7^ calculated muscle composition on preoperative computed tomography in 387 patients with localized RCC and found lower muscle mass was independently associated with decreased OS and CSS after surgery for the primary tumor. Lee et al^46^ conducted a similar study in over 600 patients with T1-T2 RCC and found sarcopenia to predict worse OS and CSS, but no significant relationship with disease progression or recurrence was identified. Increasingly, studies in patients with RCC have considered the inclusion of markers of malnutrition (ie, albumin) or inflammation (ie, modified Glasgow Prognostic Score) with sarcopenia measurements to improve prognostication and capture more RCC-specific outcomes, such as RFS.^10,32,45,51^ By including features that also reflect malnutrition and/or a patient’s inflammatory state, it is possible that sarcopenia secondary to cancer cachexia and tumor aggressiveness may be better captured,^52^ leading to the improved prediction of RCC-specific outcomes. Given these significant relationships in RCC and other malignancies, a biomarker such as the Cr/Cys-C ratio may offer a clinically practical method of screening patients most at risk for sarcopenia that would benefit from closer analysis and further intervention.

One intriguing development that may allow for intervention in patients with sarcopenia is prehabilitation programs. Prehabilitation includes preoperative medical optimization, physical exercise, nutritional supplementation, and psychological support aimed at increasing muscle mass, improving cardiorespiratory fitness, and reducing malnutrition and deconditioning.^53^ For increasing muscle mass, Hideki et al^54^ found a prehabilitation program consisting of walking, hand-grip training, and nutritionally enriched beverages in 44 patients with colorectal cancer resulted in significant increases in SMI, body weight, and skeletal muscle mass. In a cohort of patients undergoing multimodal therapy for esophageal cancer, exercise alone mitigated SMI loss compared with the control group.^55^ Although studies have considered the effectiveness of prehabilitation in patients with urologic malignancies, emphasis has primarily been on cardiorespiratory improvement rather than muscle growth, and patients with RCC have remained relatively under-explored.^56^ However, one study did examine barriers and facilitators in an exercise prehabilitation program in patients with kidney cancer,^57^ further encouraging examination of this intriguing topic.

Similar to the improved prognostication provided by combining sarcopenia with markers of inflammation or malnutrition, Cr/Cys-C may also be capturing additional elements of tumor behavior in addition to its association with sarcopenia. This was observed in our cohort, as lower Cr/Cys-C was associated with worse RCC risk scores. Along with serving as a representation of sarcopenia, which may indicate more aggressive disease, Cys-C itself has recently been examined as a prognostic factor in various malignancies.^58-60^ In RCC specifically, higher serum Cys-C has been predictive of worse disease features, OS, and RFS.^34,35,61^ Our additional analysis of Cys-C found elevated serum Cys-C levels to be associated with more aggressive RCC features and with worse OS and RFS. The potential association between Cys-C and malignancy is likely multifactorial. In addition to representing renal function, Cys-C is a cysteine protease inhibitor in all human tissues, which may attenuate the immune response to malignant cells.^34,62^ Furthermore, elevated Cys-C may correlate with inflammatory states in patients with cancer.^63,64^ Overall, increased serum Cys-C and decreased tumor tissue Cys-C appear to signal advanced stages of disease, although the role of Cys-C in RCC tumorigenesis and prognosis in this study is likely limited by sample size and warrants further study.

The Cr/Cys-C ratio as a biomarker offers clinical practicality and feasibility due to being obtained from routine laboratory values. Complex biochemical techniques, expert interpretation, and relatively small study sample sizes often limit other biomarkers and their clinical translation.^65,66^ Although promising, there were several limitations related to this work. Given the limited availability of Cys-C in our patients and the relatively recent acquisition of these values, sample size was limited. While our study indicates an association, retrospective analyses cannot establish a causal relationship. Nevertheless, the potential utility of Cr/Cys-C ratio as a prognostic factor and surrogate for sarcopenia is enticing given its simplicity and standard sarcopenia radiographic assessment methods being time intensive; expensive; and requiring expertise and specialized software. Future studies are encouraged to establish the optimal thresholds for Cr/Cys-C to ensure uniform analysis and comparison of results across different studies and patient populations. Larger studies examining the relationship between the Cr/Cys-C ratio and variance based on race, sex, and histology should be considered, as well as further examination regarding the association in patients with metastatic malignancy. Prospective validation for longer time periods is additionally needed to better understand the ability of Cr/Cys-C to predict long-term outcomes. Finally, continued exploration into prehabiltation programs and interventions to increase muscle mass should be pursued.

## Conclusion

This study identified low Cr/Cys-C ratio, as defined by lower than the median for this study cohort as independently predictive of OS and RFS in patients with RCC. Furthermore, a low Cr/Cys-C ratio was associated with sarcopenia. This work contributes to ongoing efforts for patient-specific risk stratification in RCC and other malignancies. Further validation and identification of other pragmatic and widely available, cost-effective, patient-specific risk stratification tools, such as the Cr/Cys-ratio should be pursued.

## Supplementary Material

oyad218_suppl_Supplementary_Materials

## Data Availability

The data underlying this article cannot be shared publicly due to patient privacy concerns. The data will be shared on reasonable request to the corresponding author.
