# Supplementary material for: Creatinine to Cystatin-C Ratio in Renal Cell Carcinoma: A Clinically Pragmatic Prognostic Factor and Sarcopenia Biomarker
Source: Oncologist. 2023 Aug 4;28(12):e1219–29. doi: 10.1093/oncolo/oyad218 (PMC10712910; doi:10.1093/oncolo/oyad218)
Supplement: oyad218_suppl_Supplementary_Materials [file oyad218_suppl_supplementary_materials.zip › Supplemental Tables and Figure Legends.docx]

# Supplemental Tables and Figure Legends

**Supplemental Figure 1.** Body composition analyses on L3 imaging (MRI) and corresponding Cr/Cys-C ratio for two patients. **A)** 60-year-old male with T3 renal cell carcinoma. BMI of 33kg/m^2^, cystatin-C= 1.0mg/L, creatinine= 1.1mg/dL. Considered non-sarcopenic (SMI=60.4cm^2^/m^2^) and a high Cr/Cys-C ratio (1.1). **B)** 64 year-old male with T3 renal cell carcinoma. BMI of 30kg/m^2^, cystatin-C=1.3mg/L, creatinine=0.98mg/dL. Considered sarcopenic (SMI=33.9cm^2^/m^2^) and low Cr/Cys-C ratio (0.75).

**Supplemental Table 1:** Summary of Emory and Martin et al. sex- and BMI (kg/m2)-based skeletal muscle index (cm^2^/m^2^) thresholds to diagnose sarcopenia.

**Supplemental Table 2:** Multivariable Cox hazards proportional regression analysis for overall survival and creatinine/cystatin-c ratio (n=255).

**Supplemental Table 3:** Multivariable Cox hazards proportional regression analysis for recurrence-free survival and creatinine/cystatin-c ratio (n=216; nonmetastatic patients only).

**Supplemental Table 4:** Cystatin-C levels by patient and tumor characteristics (n=255)

**Supplemental Table 5:** Multivariable Cox proportional hazards regression analyses for predictive ability of continuous cystatin-C on overall survival (n=255) and recurrence free survival (n=216, nonmetastatic only).

**Supplemental Table 6.** Correlation between creatinine to cystatin-C ratio and SMI skeletal muscle index by gender and when stratified by body mass index.
