# Supplementary material for: Creatinine to Cystatin-C Ratio in Renal Cell Carcinoma: A Clinically Pragmatic Prognostic Factor and Sarcopenia Biomarker
Source: Oncologist. 2023 Aug 4;28(12):e1219–29. doi: 10.1093/oncolo/oyad218 (PMC10712910; doi:10.1093/oncolo/oyad218)
Supplement: oyad218_suppl_Supplementary_Materials [file oyad218_suppl_supplementary_materials.zip › Supplemental table 1.docx]

| **Supplemental Table 1:** Summary of Emory and Martin et al. sex- and BMI (kg/m2)-based skeletal muscle index (cm^2^/m^2^) thresholds to diagnose sarcopenia. | | | | |
| --- | --- | --- | --- | --- |
|  | **Emory** | | **Martin** | |
| **Gender** | **BMI <30** | **BMI>30** | **BMI <25** | **BMI >25** |
| Male | <47 | <54 | <43 | <53 |
| Female | <38 | <47 | <41 | <41 |
| Abbreviations: Body mass index (BMI) | | | | |
