# Supplementary material for: Creatinine to Cystatin-C Ratio in Renal Cell Carcinoma: A Clinically Pragmatic Prognostic Factor and Sarcopenia Biomarker
Source: Oncologist. 2023 Aug 4;28(12):e1219–29. doi: 10.1093/oncolo/oyad218 (PMC10712910; doi:10.1093/oncolo/oyad218)
Supplement: oyad218_suppl_Supplementary_Materials [file oyad218_suppl_supplementary_materials.zip › Supplemental Table 2.docx]

| **Supplemental Table 2:** Multivariable Cox hazards proportional regression analysis for overall survival and creatinine/cystatin-c ratio (n=255). | | | | |
| --- | --- | --- | --- | --- |
|  | **Binary Creatinine-Cystatin-C Ratio** | | **Continuous Creatinine-Cystatin-C Ratio** | |
| **Covariate** | **Hazard Ratio**  **(95% CI)** | **P-value** | **Hazard Ratio**  **(95% CI)** | **P-value** |
| **Creatinine/Cystatin-C Ratio** |  |  |  |  |
| Below median | 2.97 (1.12-7.90) | **0.029** | - | - |
| Continuous | - | - | 0.77 (0.61-0.97) | **0.024** |
| **Age >65 years** | - | - | 1.33 (0.55-3.20) | 0.525 |
| **Gender** |  |  |  |  |
| Male | 1.07 (0.55-3.20) | 0.887 | 1.39 (0.49-3.94) | 0.538 |
| **ECOG** |  |  |  |  |
| 0 | - | - | Ref | Ref |
| ≥ 1 | - | - | 3.34 (1.14-9.78) | **0.028** |
| **Obesity** | 0.89 (0.35-2.27) | 0.808 | 0.66 (0.24-1.80) | 0.418 |
| **Diabetes** | 1.01 (0.41-2.44) | 0.989 | 1.01 (0.40-2.53) | 0.99 |
| **T-stage** |  |  |  |  |
| T1-T2 | Ref | Ref | - | - |
| T3-T4 | 8.94 (1.07-75.03) | **0.044** | - | - |
| **N-Stage** |  |  |  |  |
| N0 | Ref | Ref | Ref | Ref |
| N1 | 1.99 (0.78-5.03) | 0.148 | 2.76 (1.07-7.13) | **0.036** |
| NX | 0.62 (0.13-2.83) | 0.535 | 0.50 (0.11-2.32) | 0.373 |
| **Width of Tumor** | - | - | 1.00 (0.99-1.01) | 0.924 |
| Abbreviations: Eastern Cooperative Oncology Group (ECOG); Renal Cell Carcinoma (RCC). C-index for continuous Cr/Cys-C and overall survival=0.803. | | | | |
