# Supplementary material for: Creatinine to Cystatin-C Ratio in Renal Cell Carcinoma: A Clinically Pragmatic Prognostic Factor and Sarcopenia Biomarker
Source: Oncologist. 2023 Aug 4;28(12):e1219–29. doi: 10.1093/oncolo/oyad218 (PMC10712910; doi:10.1093/oncolo/oyad218)
Supplement: oyad218_suppl_Supplementary_Materials [file oyad218_suppl_supplementary_materials.zip › Supplemental Table 3.docx]

| **Supplemental Table 3:** Multivariable Cox hazards proportional regression analysis for recurrence-free survival and creatinine/cystatin-c ratio (n=216; nonmetastatic patients only). | | | | |
| --- | --- | --- | --- | --- |
|  | **Binary Creatinine-Cystatin-C Ratio** | | **Continuous Creatinine-Cystatin-C Ratio** | |
| **Covariate** | **Hazard Ratio**  **(95% CI)** | **P-value** | **Hazard Ratio**  **(95% CI)** | **P-value** |
| **Creatinine/Cystatin-C Ratio** |  |  |  |  |
| Ratio less than 1 | 3.31 (1.26-8.66) | **0.015** | - | - |
| Continuous | - | - | 0.77 (0.63-0.96) | **0.96** |
| **Age >65 years** | 0.85 (0.39-1.84) | 0.677 | 0.64 (0.29-1.43) | 0.275 |
| **Race** |  |  |  |  |
| White/Other | Ref | Ref | Ref | Ref |
| Black | 2.27 (0.86-5.97) | 0.096 | 2.29 (0.88-5.94) | 0.089 |
| **Gender** |  |  |  |  |
| Male | 1.40 (0.56-3.54) | 0.473 | 1.78 (0.67-4.73) | 0.245 |
| **ECOG** |  |  |  |  |
| 0 | Ref | Ref | Ref | Ref |
| ≥ 1 | 1.36 (0.33-5.55) | 0.668 | 2.20 (0.43-11.37) | 0.347 |
| **Obesity** | 0.77 (0.34-1.75) | 0.534 | 0.72 (0.32-1.63) | 0.435 |
| **Diabetes** | 1.61 (0.70-3.74) | 0.264 | 1.58 (0.69-3.60) | 0.276 |
| **Type of Nephrectomy** |  |  |  |  |
| Radical | Ref | Ref | Ref | Ref |
| Partial | 0.66 (0.13-3.30) | 0.617 | 0.67 (0.13-3.36) | 0.625 |
| **Pathologic T-Stage** |  |  |  |  |
| T1-T2 | Ref | Ref | Ref | Ref |
| T3-T4 | 2.80 (0.63-12.50) | 0.178 | 2.45 (0.54-11.13) | 0.245 |
| **Pathologic N-Stage** |  |  |  |  |
| N0 | Ref | Ref | Ref | Ref |
| N1 | 1.32 (0.51-3.46) | 0.565 | 1.16 (0.44-3.07) | 0.764 |
| Nx | 0.94 (0.30-2.99) | 0.919 | 0.85 (0.27-2.68) | 0.781 |
| **RCC Histology** |  |  |  |  |
| Non-Clear cell | Ref | Ref | Ref | Ref |
| Clear cell | 1.28 (0.46-3.56) | 0.643 | 1.41 (0.51-3.92) | 0.506 |
| **Necrosis** | 3.71 (1.24-11.11) | **0.019** | 3.84 (1.27-11.64) | **0.017** |
| **Maximum Width of Tumor** | 1.00 (0.98-1.02) | 0.912 | 1.00 (0.98-1.02) | 0.9 |
| Abbreviations: Eastern Cooperative Oncology Group (ECOG); Renal Cell Carcinoma (RCC). C-index for continuous Cr/Cys-C and recurrence free survival=0.835. | | | | |
