# Supplementary material for: Creatinine to Cystatin-C Ratio in Renal Cell Carcinoma: A Clinically Pragmatic Prognostic Factor and Sarcopenia Biomarker
Source: Oncologist. 2023 Aug 4;28(12):e1219–29. doi: 10.1093/oncolo/oyad218 (PMC10712910; doi:10.1093/oncolo/oyad218)
Supplement: oyad218_suppl_Supplementary_Materials [file oyad218_suppl_supplementary_materials.zip › Supplemental Table 4.docx]

| **Supplemental Table 4:** Cystatin-C levels by patient and tumor characteristics (n=255) | | | |
| --- | --- | --- | --- |
|  |  | **Serum Cystatin-C (mg/L)** | |
| **Covariate** | **n (%)** | **Median (IQR)** | **p-value** |
| **Age** | 64 (54-72)* | 0.395** | **<0.001** |
| **Gender** |  |  |  |
| Male | 171 (67.1) | 1.0 (0.9-1.3) | 0.841 |
| Female | 84 (32.9) | 1.0 (0.8-1.5) |  |
| **Race** |  |  |  |
| White | 161 (63.1) | 1.1 (0.9-1.3) | 0.602 |
| Black | 69 (27.1) | 1.0 (0.8-1.3) |  |
| Other | 25 (9.8) | 1.1 (0.9-1.5) |  |
| **ECOG** |  |  |  |
| 0 | 230 (90.2) | 1.0 (0.8-1.3) | 0.275 |
| ≥ 1 | 25 (9.8) | 1.2 (1.0-1.5) |  |
| **BMI (Kg/m^2^)** | 28.9 (25.8-34) | 0.034** | 0.589 |
| **Diabetes** |  |  |  |
| Yes | 88 (34.5) | 1.1 (0.9-1.6) | **0.003** |
| No | 167 (65.5) | 1.0 (0.8-1.3) |  |
| **Hypertension** |  |  |  |
| Yes | 207 (81.2) | 1.1 (0.9-1.4) | **0.001** |
| No | 48 (18.8) | 0.9 (0.7-1.0) |  |
| **Cardiovascular Disease** |  |  |  |
| Yes | 67 (26.3) | 1.2 (0.9-1.5) | **0.004** |
| No | 188 (73.7) | 1.0 (0.8-1.3) |  |
| **Preoperative Renal Function (CKD-EPI-Cr-2021)** |  |  |  |
| CKD Stage I-II | 193 (75.7) | 0.9 (0.8-1.2) | **<0.001** |
| CKD Stage III | 57 (22.4) | 1.5 (1.2-1.8) |  |
| CKD Stage IV | 5 (2) | 2.4 (2.3-2.5) |  |
| **Type of Nephrectomy** |  |  |  |
| Radical | 157 (61.6) | 1.1 (0.9-1.5) | **<0.001** |
| Partial | 98 (38.4) | 1.0 (0.8-1.2) |  |
| **Pathologic T-Stage** |  |  |  |
| T1-T2 | 116 (45.5) | 1.0 (0.8-1.2) | **<.001** |
| T3-T4 | 139 (54.5) | 1.2 (0.9-1.5) |  |
| **Pathologic N-Stage** |  |  |  |
| N0 | 161 (63.1) | 1.0 (0.8-1.3) | **0.021** |
| N1 | 30 (11.8) | 1.2 (0.9-1.6) |  |
| NX | 64 (25.1) | 1.0 (0.9-1.5) |  |
| **Pathologic M-Stage** |  |  |  |
| M0 | 216 (84.7) | 1.0 (0.8-1.3) | **<0.001** |
| M1 | 39 (15.3) | 1.3 (1.0-1.8) |  |
| **Histology** |  |  |  |
| Clear Cell | 182 (71.4) | 1.1 (0.9-1.4) | 0.145 |
| Papillary | 27 (10.6) | 1.0 (0.8-1.3) |  |
| Chromophobe | 24 (9.4) | 0.9 (0.8-1.1) |  |
| Other | 22 (8.6) | 1.1 (0.9-1.3) |  |
| **Furhman grade *(missing=17)*** |  |  |  |
| G1-G2 | 39 (17.3) | 1.0 (0.8-1.2) | **0.04** |
| G3-G4 | 187 (82.7) | 1.1 (0.9-1.4) |  |
| **Necrosis** |  |  |  |
| Yes | 142 (55.7) | 1.2 (0.9-1.5) | **0.007** |
| No | 113 (44.3) | 1.0 (0.8-1.3) |  |
| **Recurrence *(missing=36)*** |  |  |  |
| No | 207 (81.2) | 1.0 (0.8-1.3) | **0.007** |
| Yes | 48 (18.8) | 1.2 (0.9-1.5) |  |
| **Maximum Tumor Width (cm)** | 6.1 (3.8-10) | 0.035** | 0.579 |
| **Cystatin C** | 1 (0.8-1.3)* | - | - |
| *Median (IQR). **Pearson Correlation Coefficient for continuous Variables. **Abbreviations**: Eastern Cooperative Oncology Group (ECOG); Body mass index (BMI); Chronic Kidney Disease Epidemiology Collaboration 2021 Creatinine Equation without Race (CKD-EPI-Cr-2021) | | | |
