# Supplementary material for: Creatinine to Cystatin-C Ratio in Renal Cell Carcinoma: A Clinically Pragmatic Prognostic Factor and Sarcopenia Biomarker
Source: Oncologist. 2023 Aug 4;28(12):e1219–29. doi: 10.1093/oncolo/oyad218 (PMC10712910; doi:10.1093/oncolo/oyad218)
Supplement: oyad218_suppl_Supplementary_Materials [file oyad218_suppl_supplementary_materials.zip › Supplemental Table 5.docx]

| **Supplemental Table 5:** Multivariable Cox proportional hazards regression analyses for predictive ability of continuous cystatin-C on overall survival (n=255) and recurrence free survival (n=216, nonmetastatic only). | | | | |
| --- | --- | --- | --- | --- |
|  | **Overall Survival** | | **Recurrence Free Survival** | |
|  | **Hazard Ratio (95% CI)** | **P-value** | **Hazard Ratio (95% CI)** | **P-value** |
| **Cystatin C*** | 1.12 (1.01-1.24) | **0.032** | 1.09 (1.01-1.187) | **0.03** |
| **Age >65** | 0.96 (0.41-2.26) | 0.925 | 0.68 (0.31-1.49) | 0.334 |
| **Gender** |  |  |  |  |
| Male | 0.96 (0.37-2.53) | 0.936 | 1.68 (0.70-4.05) | 0.25 |
| **Black Race** | - | - | 1.08 (0.50-2.37) | 0.838 |
| **ECOG** |  |  |  |  |
| 1+ | 3.36 (1.17-9.63) | **0.024** | 2.05 (0.59-7.03) | 0.256 |
| **Obesity** | 0.58 (0.22-1.51) | 0.264 | 0.78 (0.35-1.72) | 0.539 |
| **Diabetes** | 0.89 (0.36-2.24) | 0.812 | - | - |
| **Clear Cell Histology** | 2.42 (0.30-19.17) | 0.404 | - | - |
| **Fuhrman Grade** |  |  |  |  |
| G1-G2 | Ref | Ref | - | - |
| G3-G4 | 1.69 (0.18-15.96) | 0.645 | - | - |
| **T-Stage** |  |  | - | - |
| T1-T2 | Ref | Ref | - | - |
| T3-T4 | 10.02 (1.13-88.69) | **0.038** | - | - |
| **Maximum Tumor Width** | 1.00 (0.99-1.01) | 0.964 | - | - |
| *Continuous Cystatin-C, unit=0.1mg/L. Abbreviations: Eastern Cooperative Oncology Group (ECOG). C-index for continuous Cys-C and overall survival=0.801 and recurrence free survival=0.708 | | | | |
