# Supplementary material for: Creatinine to Cystatin-C Ratio in Renal Cell Carcinoma: A Clinically Pragmatic Prognostic Factor and Sarcopenia Biomarker
Source: Oncologist. 2023 Aug 4;28(12):e1219–29. doi: 10.1093/oncolo/oyad218 (PMC10712910; doi:10.1093/oncolo/oyad218)
Supplement: oyad218_suppl_Supplementary_Materials [file oyad218_suppl_supplementary_materials.zip › Supplemental Table 6.docx]

| **Supplemental Table 6.** Correlation between creatinine to cystatin-C ratio and SMI skeletal muscle index by gender and when stratified by body mass index. | | |
| --- | --- | --- |
|  | **SMI** | **p-value** |
| Male | 0.229 | **0.003** |
| Female | 0.203 | 0.075 |
| BMI 20-25 | 0.506 | **<0.001** |
| BMI >25 | 0.283 | **<0.001** |
| Spearman correlation coefficient utilized. Abbreviations: Skeletal muscle area (SMA); Skeletal muscle index (SMI). | | |
